# Safety, prosthesis wearing time and health-related quality of life of lower extremity bone-anchored prostheses using a press-fit titanium osseointegration implant: A prospective one-year follow-up cohort study

**DOI:** 10.1371/journal.pone.0230027

**Published:** 2020-03-09

**Authors:** R. Atallah, H. van de Meent, L. Verhamme, J. P. Frölke, R. A. Leijendekkers

**Affiliations:** 1 Department of Orthopaedics, Radboud University Medical Center, Nijmegen, The Netherlands; 2 Department of Rehabilitation Medicine, Radboud University Medical Center, Nijmegen, The Netherlands; 3 Department of Oral and Maxillofacial Surgery, Radboud University Medical Center, Nijmegen, The Netherlands; 4 Department of Surgery, Radboud University Medical Center, Nijmegen, The Netherlands; 5 Radboud Institute for Health Sciences, IQ Healthcare, Nijmegen, The Netherlands; Holland Bloorview Kids Rehabilitation Hospital, CANADA

## Abstract

**Background:**

We described safety and functional one-year follow-up outcomes of individuals with lower limb amputation treated with bone-anchored prostheses using titanium press-fit osseointegration implants.

**Methods:**

All consecutive individuals treated between March 2015 and June 2018 with curved osseointegration femur implant (OFI-C) indicated for a long femoral remnant, gamma osseointegration femur implant (OFI-Y) indicated for a short femoral remnant, or osseointegration tibia implant (OTI) were eligible for this study. All adverse events were evaluated, infections were graded as follows: grade 1 and 2: low- and high-grade soft tissue infection, respectively, grade 3: deep bone infection, grade 4: septic implant failure. Functional outcome measures included prosthesis wearing time (PUS), health-related quality of life (GS), and the overall situation as an amputee (GS Q3); evaluated with the Questionnaire of persons with trans-femoral amputation (Q-TFA) before surgery and at one-year follow-up.

**Results:**

Ninety of 91 individuals were included (mean age: 54±14 yrs, 26 females); treated with 53, 16 and 21 OFI-C, OFI-Y and OTI, respectively. Soft tissue infections (grade 1: 11 events, grade 2: 10 events) were treated successfully with antibiotics except in two (OFI-C and OFI-Y), who required additional surgery due to recurrent stoma irritation and peri-stoma abscess drainage. One individual with dysvascular amputation (OTI) developed septic implant loosening and occlusion of the femoral artery resulting in a transfemoral amputation. No aseptic loosening’s occurred. One individual (OFI-Y) required stoma surgical refashioning due to soft tissue redundancy. At baseline mean ±SD and median (25^th^ to 75^th^ PCTL) Q-TFA PUS and GS were 52±39, 52(7–90) and 40±19, 42(25–50) and improved significantly to 88±18, 90 (90–100) and 71±15, 75 (67–83) at one-year follow-up. The GS Q3 improved over time.

**Conclusion:**

Titanium osseointegration implants can be safely used within a one-year follow-up period. The performance improved compared to the use of a socket-suspended prosthesis.

## Introduction

Bone-anchored prostheses (BAP) using an osseointegration implant (OI) are a suitable alternative for individuals with amputations experiencing pain, pressure sores, and mobility restrictions related to the use of socket-suspended prostheses (SSP).[1]The advantage of an OI is that it provides a direct skeletal attachment for an artificial leg.[2] This results in a more physiological and stable prosthetic control, osseoperception, improved walking, and sitting conditions as well as eliminating the socket-residuum interface with all its associated problems.[3–6] Currently, there are two different OI systems commercially available.[7] The oldest, with the longest follow up evaluations, is the titanium screw fixation system developed by the Swedish Brånemark group and available as Osseointegrated Protheses for the Rehabilitation of Amputees Implant system (OPRA) manufactured by Integrum AB Sweden.[8] A second relatively more recent designed OI system is the press-fit fixation system developed and used by the German/Dutch/Australian osseointegration groups available as the Integral leg prosthesis (ILP)/Osseointegrated femur or tibia prosthesis (OFP-OTP)/Osseointegration prosthetic limb (OPL, type A-D) implant systems manufactured by Eska Orthopedics GmbH, Germany/OTN Implant BV, Netherlands/Permedica SPA, Italy; respectively.[6, 9–12] All afore mentioned OI’s are of a titanium alloy with exception of the ILP which is made of a chromium-cobalt-molybdenum alloy. The press-fit OI system is adopted from the uncemented total hip implants in which the stem has a rough macroporous surface to provide solid and fast osseointegration by means of bony ingrowth.[13] Therefore, the total treatment period for press-fit implants is currently less time-consuming than for screw type implants, meaning that the period until full weight bearing is much shorted when treated with a press-fit implant.[1, 6, 14] A recent systematic review of the safety of BAP showed a slightly better femoral OI survival for press-fit implants compared to screw implants.[15] Several studies have shown favourable performance data when comparing BAP to SSP leading to increased level of function, activity, and health-related quality of life.[1, 4–6, 16]

Previous risk-benefit studies of BAP using a press-fit OI have predominantly included selected individuals with transfemoral amputation treated with the curved press-fit osseointegration femur implant (OFI-C), both with a chromium-cobalt-molybdenum and a titanium alloy.[15] Currently almost half of the candidates referred to our center for OI treatment have either short femoral remnants or a transtibial amputation. For individuals with transfemoral amputation with short femoral remnants and individuals with transtibial amputation, a gamma press-fit osseointegration femur implant (OFI-Y) and press-fit osseointegration tibia implant (OTI) is used, respectively. Safety and performance data focusing on the OFI-Y and OTI are scarce. There are only a few case series with short follow up that report on safety and performance data of individuals with transtibial amputation.[5, 17–19] For further expansion of the application of BAP using OI in the broader population of individuals with a lower extremity amputation, insight in the risk/benefit ratio of the OFI-Y and OTI is needed; especially when compared to the risk/benefit ratio of the more widely used OFI-C.

The aim of this one-year follow-up study was to present the adverse events, prosthesis wearing time and health-related quality of life of OI’s made of a titanium alloy both in general and stratified by OI type (OFI-C, OFI-Y, and OTI).

## Materials and methods

### Study design

This article presents one-year follow-up data of an on-going cohort study. The performance data was prospectively collected as part of a larger longitudinal study.[20] One year follow-up results of a subcohort were published earlier.[5] The Strengthening the Reporting of Observational studies in Epidemiology (STROBE) statement was followed for the preparation of the manuscript to ensure methodological quality provided in S1 Appendix.[21] The study was conducted according to the principles of the Declaration of Helsinki (64^th^ version, 19-10-2013). The protocol of this study (registration number 2014/196) was approved by the Ethics Committees of Radboudumc.

### Participants

All consecutive individuals who received a titanium press-fit OFI-C (OFP and OPL type A), OFI-Y (OFP) or OTI (OTP) at the Radboud university medical center (Radboudumc), between March 2015 and June 2018, were eligible for this study. During this period a small subset of individuals were treated with other types of implants (ILP and OPL type B) but these were excluded because a) we implanted only small numbers or b) the implant was made of chromium-cobalt-molybdenum alloy. Individuals are eligible for an OI if the primary amputation is congenital or due to a trauma, tumor resection, dysvascular disease, infection, or other causes such as joint replacement infections. Additionally they have to meet the inclusion and exclusion criteria as presented in Table 1, with the inclusion criteria being based on certain items of the Q-TFA.[6] Prior to the inclusion a written informed consent was obtained from all participants.

**Table 1:** Inclusion and exclusion criteria.

Inclusion criteria: OI implant is indicated when at least one item is answered yes.	Exclusion criteria
The prosthesis is used less than 50 hours per week due to socket-related problems	Severe diabetes (including a medical history of multi-organ failure)
The prosthesis restricts walking distance: less than 2 km (with or without walking aids)	Systemic/local infection
The prosthesis is considerably unreliably attached during daily activities	Age <18 (immature bone)
The prosthesis is considerably uncomfortable to sit down	Bone deformity, -dysplasia, -metabolic disorders
The prosthesis causes sores, chafing, or skin irritation	Radiotherapy on residual limb within 3 months before OI surgery
The prosthesis considerably causes troubles by heat/sweating during hot weather	Chemotherapy within 3 months before OI surgery
The problems experienced with current prosthesis are considerable	Immunosuppressive drugs use

OI: Osseointegrated implant.

### Patient selection

The patient selection was performed with a multidisciplinary team including an orthopedic (trauma) surgeon, rehabilitation physician, and a physical therapist. Prior to their visit, the candidates completed the Questionnaire for Persons with a Transfemoral Amputation (Q-TFA) and underwent plain X-ray radiologic examination of the femoral or tibial remnant and calibrated total view of both lower extremities. A computed tomography (CT) scan was performed in individuals with a tibial amputation or in individuals with short femoral remnants as further detailed below. General information was given in a group presentation and informed consent was obtained individually by the surgeon. Three months after the general intake, mutual agreement with informed consent for the treatment was achieved based on in- and exclusion criteria, medical history, physical examination, and radiology results. Candidates who revealed unrealistic, expectations of their future functioning with a bone-anchored prosthesis, were referred to a clinical psychologist for discussion and adjustment of expectations. Candidates with a medical history of peripheral vascular disease as the cause of amputation were additionally screened by a vascular surgeon assessing the presence of femoral artery pulsations in the groin as well as skin perfusion oxygen pressure and evaluating duplex ultrasonography of the limb. A transcutaneous oxygen pressure less than 40 mmHg, measured at the tip of the stump, was used as an exclusion criterion for osseointegration surgery. Transcutaneous oxygen was measured with the Precise 8001 (MediCap Homecare GmbH, Germany).

### Surgery and implant details

**Fig 1:**
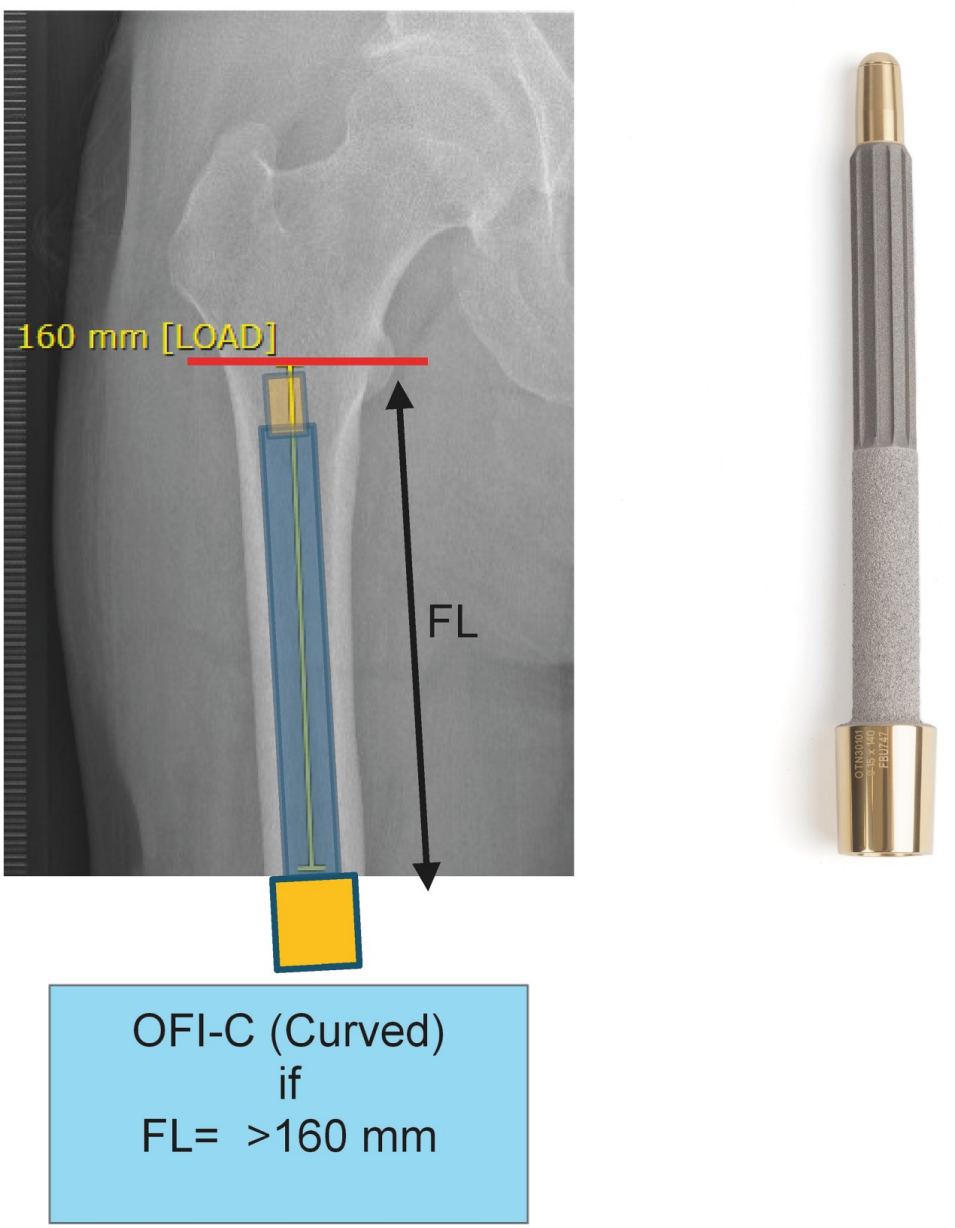
Preoperative planning and measurement of femoral remnant in OFI-C. OFI-C: Osseointegration Femur Implant curved type, FL: Femur length.

**Fig 2:**
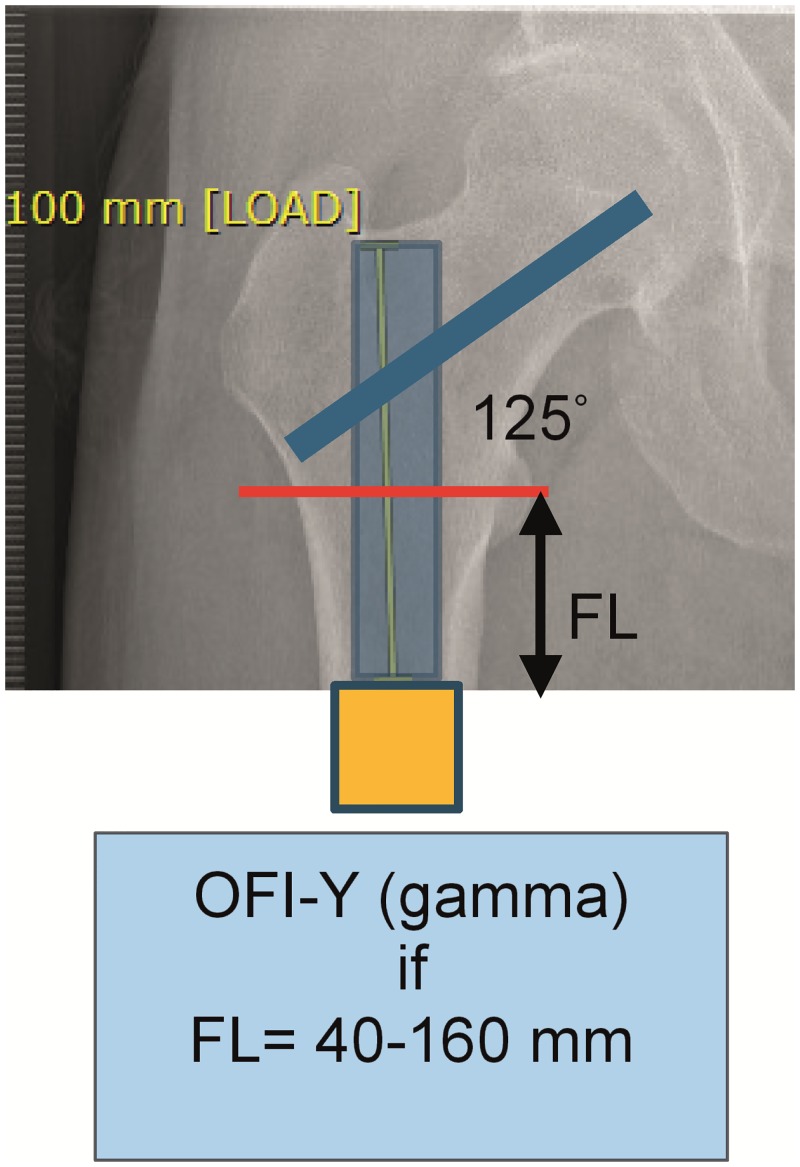
Preoperative planning and measurement of femoral remnant in OFI-Y. OFI-Y Osseointegration Femur Implant Gamma type, FL: Femur length.

**Fig 3:**
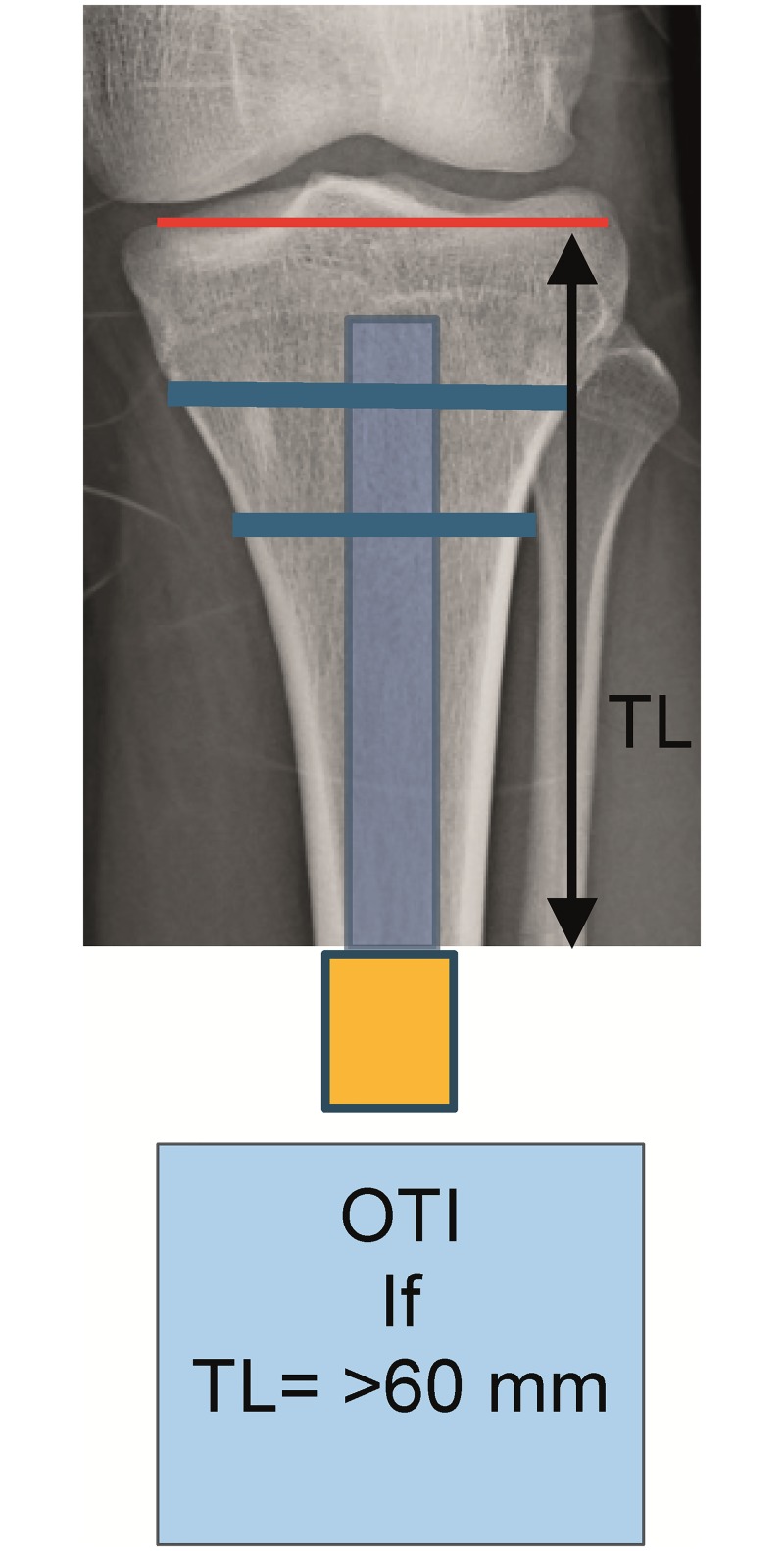
Preoperative planning of tibial remnant in OTI. OTI: Osseointegration Tibia implant, TL: Tibia length.

**Fig 4:**
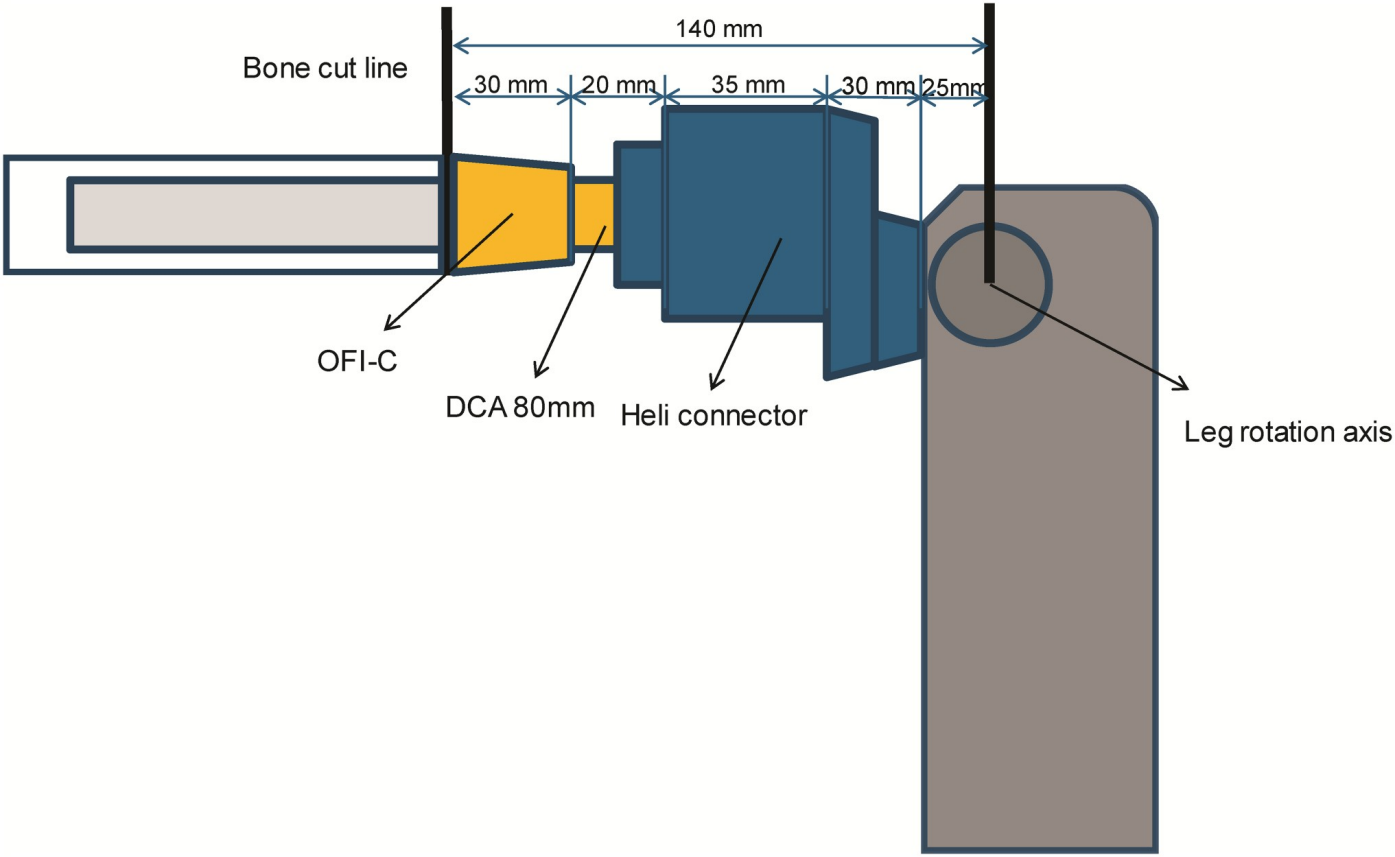
Schematic presentation of presurgical planning OFI-C. OFI-C: Osseointegration femur implant curved type, DCA: Dual cone adapter, Heli connector produced by OTNInnovations.

**Fig 5:**
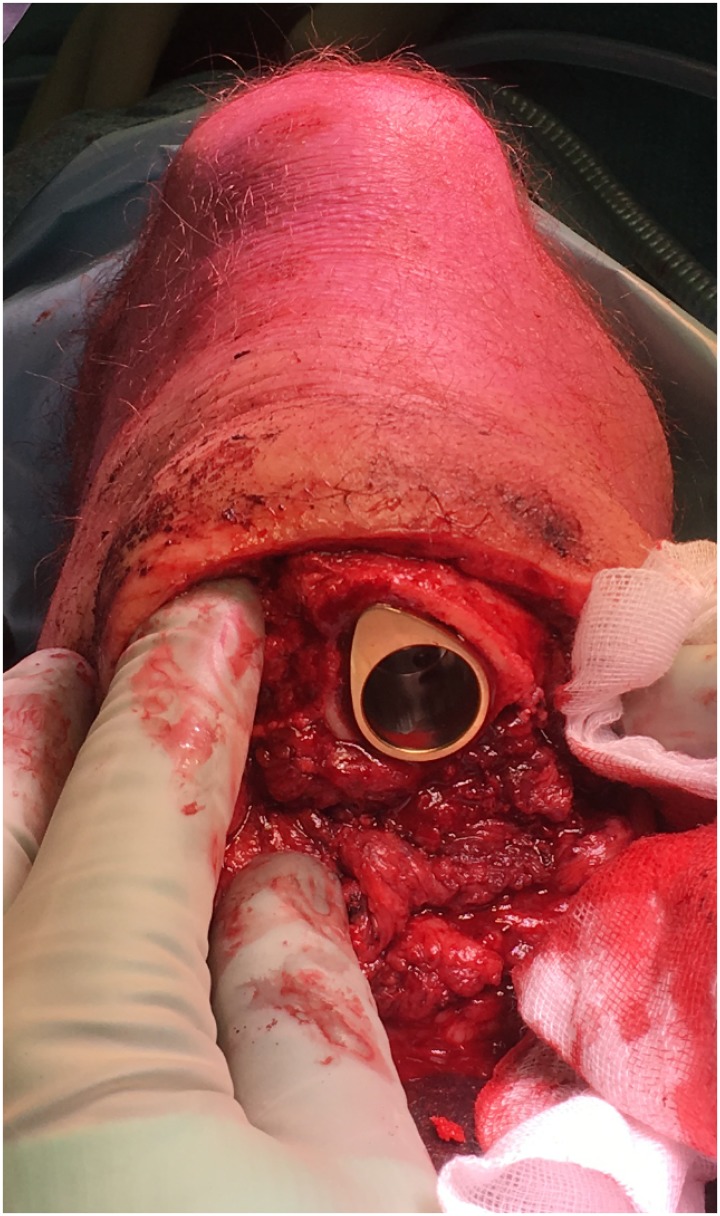
Seal of intramedullary canal by drop-like shaped implant.

**Fig 6:**
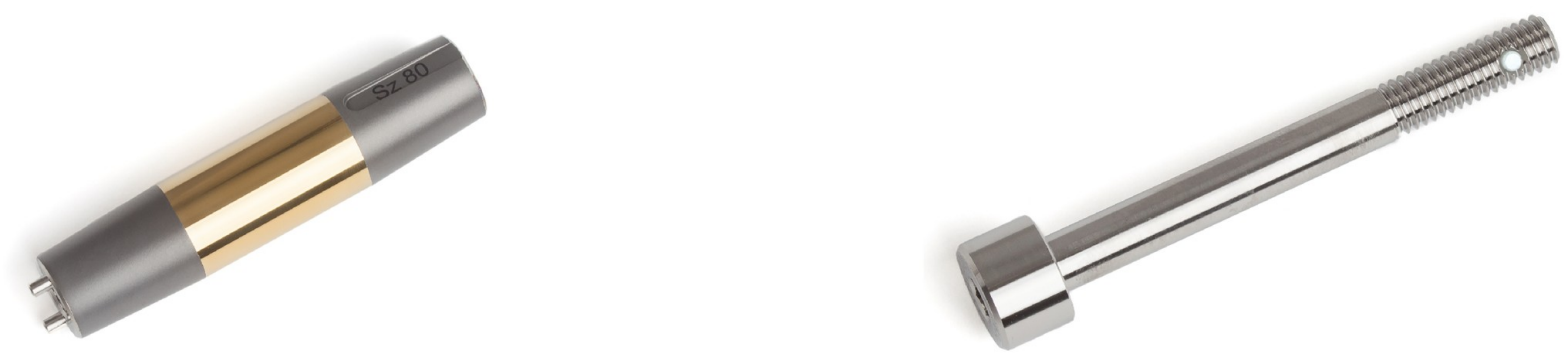
Dualcone adapter and internal locking screw.

**Fig 7:**
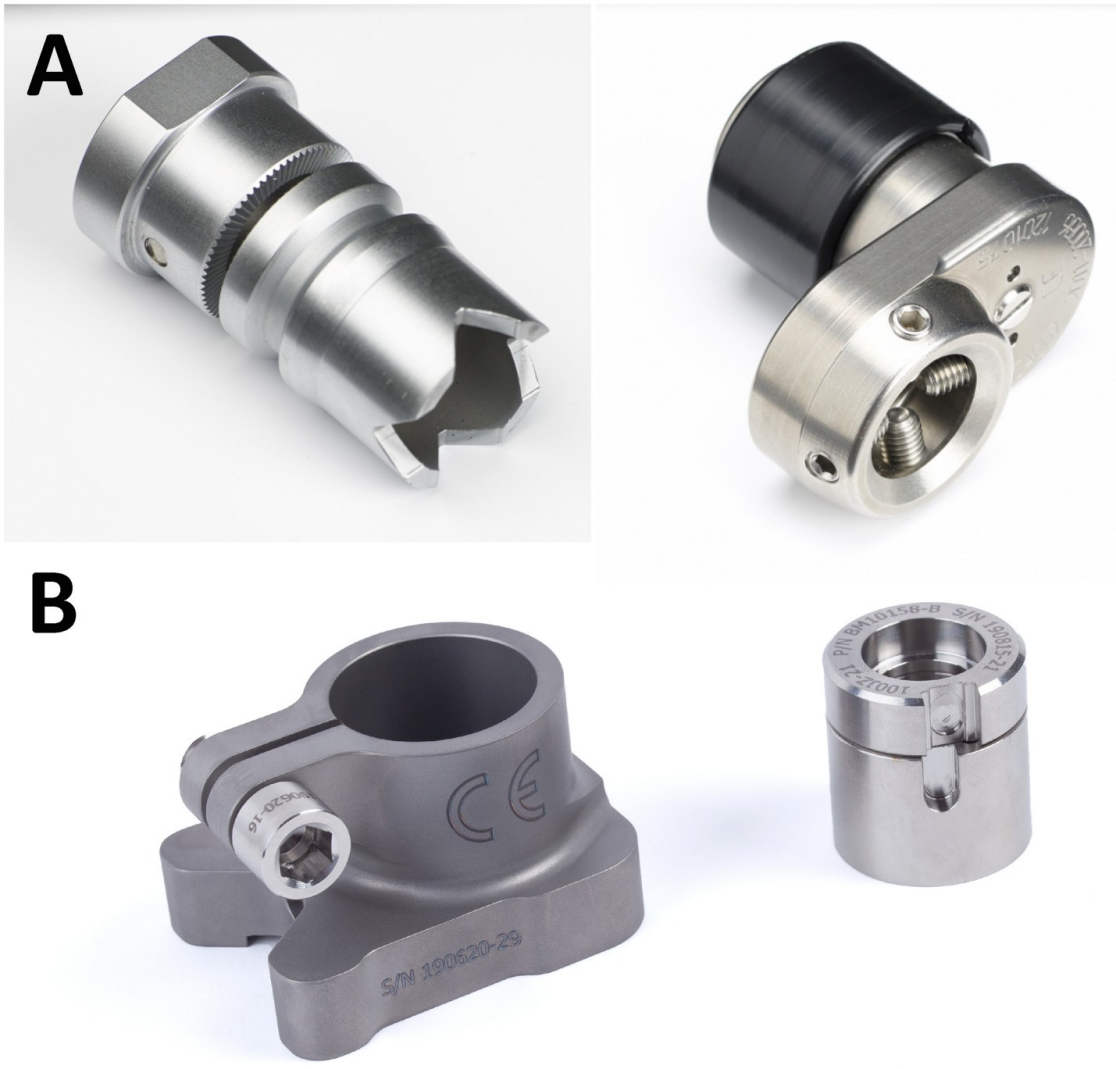
Osseointegration implant connector (A: OTN connector, B: OPL/Hermle connector).

Patients included for OI surgery were scheduled for standard two stage surgery with an interval of 6–8 weeks in between. In selected cases the surgery was performed as a single stage approach, most often necessary when there was insufficient skin to cover the tip of the intramedullary component of the OI. For patients who opted for an OFI the minimum length of the femoral remnant is 160mm or 40mm below the mid lesser trochanteric line in case of an OFI-C or OFI-Y, respectively (Figs 1 and 2). For an OTI the minimum length of the tibial remnant is 60mm below the tibial plateau (Fig 3). There is also a maximum length of the femoral and tibial remnant for prosthetic parts to be able to fit properly to the dual cone adapter (DCA) using an OI connector (Fig 4). Both the OFI-C and OFI-Y contain a cylindrical distal portion of the intramedullary stem which can adequately seal off the intramedullary canal of the diaphyseal portion of the femur. The OTI differs from the OFI as its distal portion contains a drop-like shape to provide optimal sealing of the tibial intramedullary space (Fig 5). The OI is a modular system comprising of an intramedullary stem, either with or without an additional lag/locking screw; and a DCA with an internal locking screw (Fig 6). The OI is then connected to the prosthetic parts via an osseointegration implant connector (Fig 7). Additional implant details can be found in Table 2 and additional information regarding the pre-surgical planning, the surgical procedure, the components and the prosthetic alignment can be found in S2 Appendix.

**Table 2:** Implant details.

	Length (mm)	Shape	Material	Surface	Rotational stability
OFI-C	140 or 160	Curved (radius 2000mm)	Titanium	Coating plasma sprayed titanium	Proximal longitudinal flutes stem
OFI-Y	80 to 140	Straight with 125° lag screw hole	Titanium	3D lattice structure 1mm	One lag screw hole
OTI	60 to 100	Straight with drop-like distal portion	Titanium	3D lattice structure 1mm	Two locking screw holes

OFI-C: Osseointegration Femur Implant curved type, OFI-Y: Osseointegration Femur Implant Gamma type, OTI: Osseointegration Tibia implant, mm: millimeters, 3D: 3 dimensional.

### Rehabilitation and aftercare

Rehabilitation started one week after the second OI surgery, or 3 weeks after single stage OI surgery, with loading the full-length prosthesis based on pain (numeric grading score 0–10: aim score <5) building up to full bodyweight. [5, 22]The rehabilitation was given in group sessions twice per week with sessions of two hours each and a total duration of 4 weeks or 11 weeks for tibial and femoral BAP, respectively. Follow-up visits including radiologic examination and performance tests were scheduled prior to stage 1 surgery and one year after stage 2 surgery.

### Adverse events

The adverse events during the first year after OI surgery were retrospectively extracted from the participants’ medical files. All adverse events related to OI surgery were reported and were included in the database such as: infection, bone/implant breakage, implant aseptic loosening (radiographic evidence of loosening with the absence of infection), stoma redundant tissue (soft-tissue surplus around the transcutaneous connection), and death as well as the necessary treatment. Infections were graded using the classification described by Al Muderis et al., grade 1 (low-grade soft tissue infection), 2 (high-grade soft tissue infection), 3 (deep bone infection), 4 (septic implant failure); which can also be found in S3 Appendix.[9] Adverse events were graded severe (Grade 3 and 4 infection, implant breakage, aseptic loosening, bone fracture, death) or minor (Grade 1 and 2 infection, stoma redundant tissue).[5]

### Performance measures

Prior to OI surgery, each participant underwent pre-operative evaluation using their SSP and the evaluation with BAP was performed twelve months after the second surgery. Prosthesis wearing time was scored using the Q-TFA prosthetic use score (range 0–100). Health-related quality of life was measured with the Q-TFA global score (range 0–100).[23] The global score is not applicable for patients who are non-prosthetic users.[16] Therefore the third question of the global score “How would you summarize your overall situation as an amputee?” with five response options (extremely poor, poor, average, good, extremely good) was specifically used, which is also applicable for non-prosthetic users. The Q-TFA questionnaires were sent electronically to the patients using a web-based database (Castor EDC, Amsterdam the Netherlands) prior to their visit and were either in Dutch or English.

## Statistical analysis

All safety and performance data were stored and processed using a web-based database (Castor EDC, Amsterdam the Netherlands). Demographics and participant characteristics are presented descriptively. Categorical data was presented as exact numbers. Percentages were calculated for the various levels. For continuous data, means and standard deviations were calculated for normally distributed variables. For data not-normally distributed median, 25^th^ and 75^th^ percentile were used. Q-TFA PUS and GS were presented in means, standard deviations as well as median and 25^th^ and 75^th^ percentile. Changes between pre- and post OI surgery were analyzed using a complete case analysis for both the entire cohort and stratified by OI type (OFI-C, OFI-Y, and OTI). Normally distributed continuous outcomes were statistically analyzed with a paired student-t test (Q-TFA GS). Not-normally distributed continuous were analyzed using the Wilcoxon signed-rank test (Q-TFA PUS). To compare infection rates between the 3 subgroups of different sizes we calculated the infection/implant-year ratio as described by Tillander et al.[24]

A p-value of <0.01 was considered statistically significant. A p-value of <0.01 was used to reduce the risk of type I errors due to multiple testing. All analyses were performed using SPSS v23 (SPSS Inc., Chicago, Illinois, United States).

## Results

Between March 2015 and June 2018, 90 consecutive individuals met the in- and exclusion criteria as indicated in Table 1. These included 66 transfemoral (3 bilateral), 20 transtibial (1 bilateral), 3 through-knee amputations, and 1 without an amputation but with a non-functional leg which was covered with split-skin grafts due to a trauma and therefore was not eligible for a SSP (94 OI’s). One additional patient was implanted with a titanium OI (OTI) within the inclusion period but was excluded from the study because of severe diabetes. The overall and OI-specific patient baseline characteristics and amputation and surgical details are summarized in Table 3. The cohort of 90 individuals had an average age of 54 years (range 20–86) and included 26 females. The average age at primary amputation was 40 years and average age at OI implantation was 54 years. The cause of primary amputation was; trauma n = 50, dysvascular n = 12, infection n = 12, tumor n = 8, congenital n = 3, other n = 8. Of the 94 OI’s the number of implanted OFI-C, OFI-Y, and OTI was 55, 17, and 22 respectively. The median applied OFI-C diameter was 16mm and all OFI-C had a length of 160mm. The median applied OFI-Y diameter was 21mm with a median length of 140mm and the median OTI diameter was 21.5mm with a median length of 90mm.

**Table 3:** Patient demographics.

	Total (N = 90)	OFI-C (N = 53)	OFI-Y (N = 16)	OTI (N = 21)
Patient demographics				
- Sex*				
• Male	64 (71%)	36 (68%)	13 (81%)	15 (71%)
• Female	26 (29%)	17 (32%)	3 (19%)	6 (29%)
- Age (y)				
• At amputationϮ	40 ± 18	44 ± 19	31 ± 17	37 ± 16
• At implantationϮ	54 ± 14	57 ± 14	50 ± 15	48 ± 13
• Interval between amputation and implantation (y)+	8 [4 to 8]	6 [4 to 17]	17 [8 to 28]	6 [3 to 13]
- Country of origin				
• Netherlands	82 (91%)	48 (91%)	14 (88%)	20 (95%)
• United Kingdom	3 (3%)	2 (4%)	0 (0%)	1 (5%)
• United States of America	1 (1%)	0 (0%)	1 (6%)	0 (0%)
• Norway	1 (1%)	1 (2%)	0 (0%)	0 (0%)
• Italy	1 (1%)	0 (0%)	1 (6%)	0 (0%)
• Aruba	1 (1%)	1 (2%)	0 (0%)	0 (0%)
• Serbia	1 (1%)	1 (2%)	0 (0%)	0 (0%)
Baseline amputation characteristics				
- Level* (per limb: N = 93^)				
• TFA	69 (74%)	52 (95%)	17 (100%)	NA
• TTA	21 (23%)	NA	NA	21 (100%)
• TK	3 (3%)	3 (6%)	NA	NA
- Side* (N = 89^)				
• Left	42 (47%)	22 (42%)	7 (44%)	13 (65%)
• Right	43 (48%)	29 (55%)	8 (50%)	6 (30%)
• Bilateral	4 (4%)	2 (4%)	1 (6%)	1 (5%)
- Cause (per limb: N = 93^)				
• Trauma	50 (54%)	25 (46%)	11 (65%)	14 (67%)
• Dysvascular	12 (13%)	9 (16%)	0 (0%)	3 (14%)
• Infection	12 (13%)	9 (16%)	1 (6%)	2 (10%)
• Tumor	8 (9%)	5 (9%)	3 (18%)	0 (0%)
• Congenital	3 (3%)	1 (2%)	2 (12%)	0 (0%)
• Other	8 (9%)	6 (11%)	0 (0%)	2 (10%)
Surgical details (per implant: N = 94)				
• Single stage*	17 (18%)	6 (11%)	4 (24%)	7 (32%)
• Two stage*	76 (81%)	49 (89%)	13 (77%)	14 (64%)
• Primary amputation + Implantation OI in one stage*	1 (1%)	0 (0%)	0 (0%)	1 (5%)
Implant characteristics (per implant: N = 94)				
• Width (cm)+	NA	16 [15 to 17]	21 [18 to 23]	21.5 [19 to 23]
• Length (cm)+	NA	160 [160]	140 [95 to 163]	90 [79 to 106]

* The values are given as the number of patients/implants with the percentage in parentheses.

^Ϯ^ The values are given as the mean and standard deviation.

^+^ The values are given as the median and 25^th^ and 75^th^ percentile.

^One individual/limb less at baseline due to not having underwent amputation yet. Y: years, NA: Not applicable, N: Participants, TFA: Transfemoral amputation, TTA: Transtibial amputation, TK: Through knee amputation, Cm: centimeters. OFI-C: Osseointegration femur implant curved type, OFI-Y: Osseointegration femur implant gamma type, OTI: Osseointegration tibia implant

**Fig 8:**
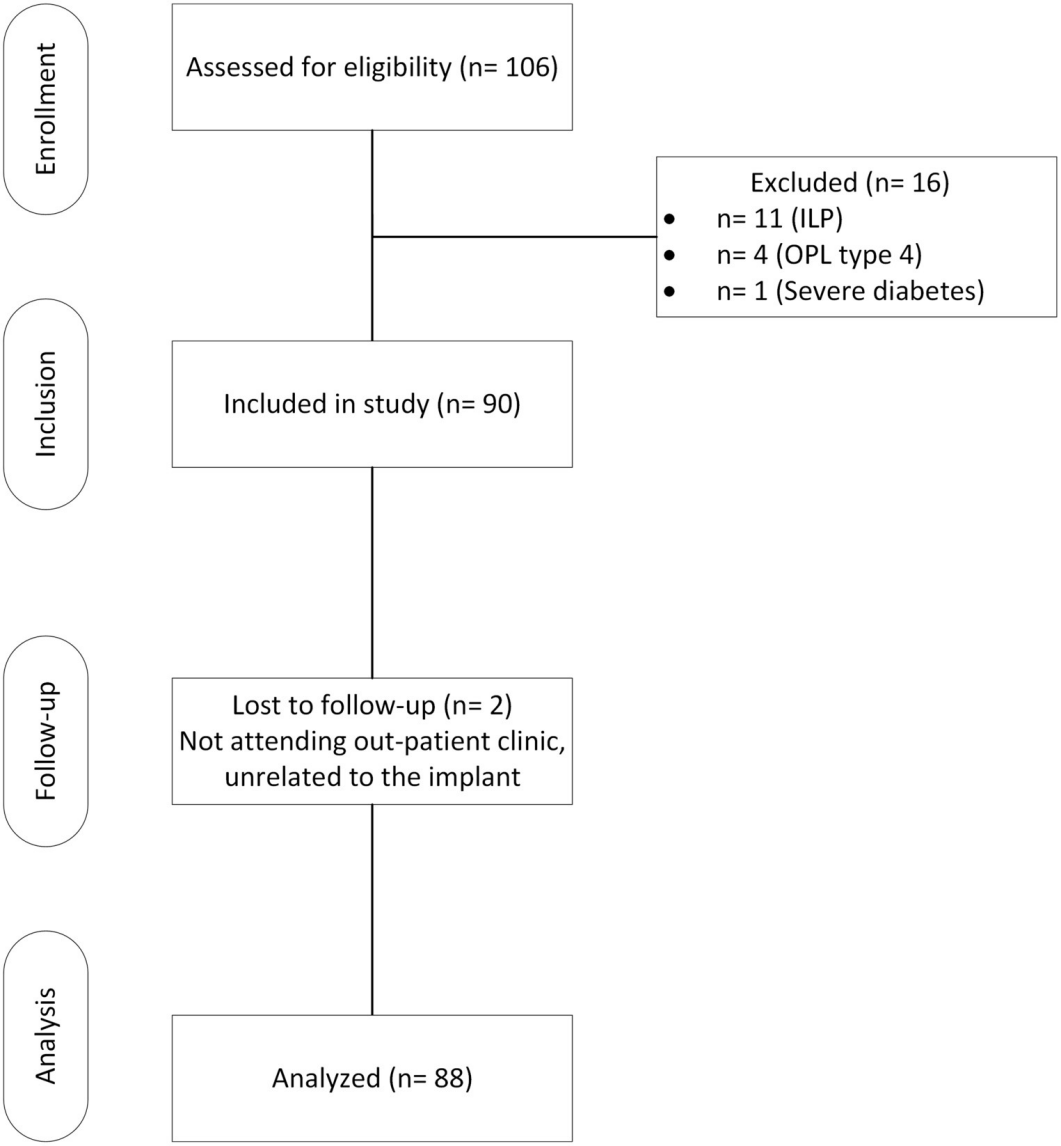
Participant flow diagram. ILP: Integral leg prosthesis, OPL type B: Osseointegration prosthetic limb type B.

Two patients were lost to follow-up (OTI and OFI-C), who did not attend the outpatient clinic at 1 year follow-up due to reasons unrelated to the BAP (Fig 8).

### Severe adverse events

In 88 individuals (92 OI’s), one individual with an OTI (1%) developed a grade 4 septic implant loosening resulting in subsequent transfemoral amputation. The primary cause of amputation was chronic arterial occlusive disease and at inclusion patient had palpable femoral pulsations. One month after OI surgery he developed a complete femoral artery occlusion. No grade 3 or aseptic implant loosening occurred during the follow-up time period. No intramedullary stem breakage occurred. Four breakages of the transcutaneous component (DCA) of the BAP occurred; three individuals (two with OFI-Y and one with OTI) had a breakage of the distal taper and one individual with OFI-C had broken weakpoints of the DCA. All broken DCAs were successfully replaced in an outpatient clinic setting. Two individuals experienced bone fractures, one hip neck fracture after a fall (OFI-C) which was treated successfully with dynamic hip screw osteosynthesis and one lumbar vertebra fracture after a fall (OFI-C) which was treated non-operatively with a brace. The adverse events are summarized in Table 4.

**Table 4:** Adverse events.

Adverse events	Total cohort (n = 88)%	OFI-C (n = 52)%	OFI-Y (n = 16)%	OTI (n = 20)%
Infection				
• Grade 1	11 (13%)	4 (8%)	3 (19%)	4 (20%)
• Grade 2	10 (11%)	4 (8%)	2 (13%)	4 (20%)
• Grade 3	-	-	-	-
• Grade 4	1 (1%)	-	-	1 (5%)
Bone breakage	2 (2%)	2 (4%)	-	-
Implant breakage				
• Intramedullary stem	-	-	-	-
• DCA	4 (5%)	1 (2%)	2 (13%)	1 (5%)
Aseptic loosening	-	-	-	-
Stoma redundant tissue	1 (1%)	-	1 (6%)	-
Death	-	-	-	-

N: participants, OFI-C: Osseointegration femur implant curved type, OFI-Y: Osseointegration femur implant gamma type, OTI: Osseointegration tibia implant, DCA: Dual cone adapter. - = 0 (0%)

### Minor adverse events

Eleven of the 88 individuals (13%) developed a grade 1 soft tissue infection and 10 individuals (11%) developed grade 2 soft tissue infection. All grade 1 and 2 soft tissue infections were pin-track infections and occurred in the first months after OI surgery. All grade 1 infections were successfully treated with oral antibiotics (grade 1A). Grade 2 infections were treated successfully with oral antibiotics in 8 cases (grade 2A), while two individuals required additional soft tissue surgery (grade 2C), due to either recurrent irritation and infection or a peri-stoma abscess (OFI-C n = 1, OFI-Y n = 1). Antibiotics used were floxacillin, amoxicillin/clavulanic acid or ciprofloxacin. Five individuals that underwent two stage OI surgery experienced wound infections after stage 1 and therefore, step two of the surgery was performed earlier (OFI-C n = 2, OFI-Y n = 2, OTI n = 1); on average 2.5 weeks after stage 1. In one individual step two was performed earlier due to pain, operative swabs taken did not show any growth of bacteria. The number of individuals with soft tissue infections related to OFI-C, OFI-Y, and OTI were: 8, 5, and 8, respectively. No individuals experienced multiple events of infections of the same grade. Correcting for the differences in numbers per group by using the infection/implant-year ratio this amounts to a ratio of 8/54 (infections per implant with 1 year follow-up: 14.8%), 5/17 (29.4%) and 9/21 (42.9%) for the OFI-C, OFI-Y, and OTI, respectively. One individual, treated with an OFI-Y, required soft tissue surgery due to redundancy of soft tissue. Other reported adverse events included; pulmonary embolism after stage 1 OFI-C implantation successfully treated with anticoagulants (n-1), transient knee pain after OTI (n = 1), transient groin pain after OFI-C (n = 1), and distal femoral heterotopic bone formation (OFI-C) in one patient that used Aclasta intravenously for the treatment of glucocorticoid-induced osteoporosis. One patient (OFI-C) developed transient nausea, hypertension, and pain at 5 months after OI surgery but these complaints disappeared suddenly and inexplicable with few minor adaptations of the prosthetic alignment.

### Performance

Sixteen of the 90 individuals (18%) were non-prosthetic users at baseline (OFI-C: 8/53 (16%), OFI-Y: 8/16 (50%), OTI: 0/21 (0%)). At follow-up, 87 individuals were ambulators using their BAP; including all individuals that were non-prosthetic users at baseline, while there was missing data for 3 individuals (loss to follow-up n = 2 and septic implant loosening n = 1).

One individual underwent amputation and implantation in a single setting and thus had missing Q-TFA data at baseline. The performance data for the entire cohort and stratified by implant are summarized in Tables 4 and 5. Both the PUS and the GS increased significantly at follow-up for the entire cohort and when stratifying per OI type. An improvement in the overall situation as an amputee is seen when comparing baseline to one year follow-up since the percentage of participants who scored “good” or “very good” increased over time both for the entire cohort and when stratifying per OI type (Table 6).

**Table 5:** Performance outcomes (Q-TFA prosthetic use score and global score).

	Baseline (T0)		1 year FU (T1)		Difference (T1 –T0) Mean ± SD	p-value+
	Mean ± SD	Median (25th to 75th PCTL)	Mean ± SD	Median (25th to 75th PCTL)		
Q-TFA PUS						
Total cohort (n = 87)*	52 ± 39	52 [7 to 90]	88 ± 18	90 [90 to 100]	NA	<0.01+
OFI-C (n = 52)*	59 ± 37	71 [25 to 90]	86 ± 19	90 [76 to 100]	NA	<0.01+
OFI-Y (n = 16)*	31 ± 41	5 [0 to 69]	93 ± 12	100 [90 to 100]	NA	<0.01+
OTI (n = 19)*	50 ± 39	52 [10 to 90]	87 ± 21	100 [90 to 100]	NA	<0.01+
Q-TFA GS						
Total cohort (n = 70)*	40 ± 19	42 [25 to 50]	71 ± 15	75 [67 to 83]	32 ± 22	<0.01^
OFI-C (n = 44)*	42 ± 19	42 [25 to 50]	67 ± 16	75 [58 to 75]	25 ± 19	<0.01^
OFI-Y (n = 8)*	31 ± 18	42 [12 to 42]	79 ± 10	75 [75 to 83]	48 ± 17	<0.01^
OTI (n = 18)*	38 ± 21	33 [23 to 54]	79 ± 11	79 [75 to 83]	41 ± 24	<0.01^

* Number of individuals included in the analysis,

^+^ Calculated using the Wilcoxon signed-rank test,

^Calculated using the paired student-t-test, Q-TFA: Questionnaire for persons with a Transfemoral amputation, PUS: prosthetic use score, GS: Global score, OFI-C: Osseointegration femur implant curved type, OFI-Y: Osseointegration femur implant gamma type, OTI: Osseointegration tibia implant, PCTL: percentile, N: Participants, FU: Follow-up, NA: not applicable.

**Table 6:** Overall situation as an amputee (Q-TFA Global score question 3).

Overall situation (Q-TFA GS Q3)	Total cohort (n = 87/90)*		OFI-C (n = 52/53)*		OFI-Y (n = 16/16)*		OTI (n = 19/21)*	
	Baseline	1 year FU	Baseline	1 year FU	Baseline	1 year FU	Baseline	1 year FU
Extremely poor	3 (3%)	1 (1%)	1 (2%)	1 (2%)	1 (6%)	0 (0%)	1 (5%)	0 (0%)
Poor	25 (29%)	0 (0%)	16 (31%)	0 (0%)	2 (13%)	0 (0%)	7 (37%)	0 (0%)
Average	32 (37%)	17 (20%)	18 (35%)	14 (27%)	7 (44%)	1 (6%)	7 (37%)	2 (11%)
Good	20 (23%)	55 (63%)	13 (25%)	29 (56%)	4 (25%)	11 (69%)	3 (16%)	15 (79%)
Extremely good	7 (8%)	14 (16%)	4 (8%)	8 (15%)	2 (13%)	4 (25%)	1 (5%)	2 (11%)

Q-TFA GS Q3: Questionnaire for persons with a Transfemoral amputation global score question 3. N: participants, FU: follow-up, OFI-C: Osseointegration femur implant curved type, OFI-Y: Osseointegration femur implant gamma type, OTI: Osseointegration tibia implant,

* Number of individuals included without missing data out of total.

## Discussion

Taking the short follow-up into account our results indicate that OI surgery is a safe treatment option for individuals with a lower extremity amputation, regardless the level of amputation, who experience complaints with SSP. The most prevalent adverse events are transient soft tissue adverse events that are fairly easy to handle with either more intensive stoma care and/or antibiotics.

The benefits, with regard to prosthesis wearing time and quality of life, greatly outweigh the drawbacks they encounter. Individuals with an OFI-Y showed the largest improvement in the PUS at follow-up probably because 50% of individuals with OFI-Y were non-prosthetic users at baseline. This result clearly identifies a specific group with high level transfemoral amputation that benefits greatly from BAP.

We assumed that the incidence of aseptic loosening would possibly be higher in individuals treated with an OFI-Y or OTI due to differences in fixation, in which the OFI-Y/OTI fixate in meta- and epiphyseal bone while the OFI-C has a diaphyseal fixation. The OFI-Y and OTI are also much shorter which would result in a smaller surface area for osseointegration. To compensate for the smaller osseointegration area the OFI-Y and OTI were designed with a 3D lattice structure, which creates a 3.7 times larger surface area when compared to an implant without a 3D lattice structure. In this study no aseptic implant loosening occurred which might indicate that OI’s with short implant lengths provided with the correct mesh surfaces and additional locking screws may lead to adequate integration in short femoral or tibial remnants. This finding creates favorable perspectives for individuals with short residual limbs as this group often experiences the most problems with socket-suspended prostheses, when looking at our own clinical experience.

Differences in shape and volume of the stump might have influenced soft tissue adverse events in this study. In our experience individuals treated with an OFI-Y most often have excess soft tissue and therefore might need soft tissue refashioning more often. In our experience individuals with a transtibial amputation often have limited excess of and therefore need single stage surgery more often. Single stage surgery was performed for OTI, OFI-Y and OFI-C in 32%, 24%, and 11%, respectively. Individuals with OTI experienced the most infectious soft tissue adverse events, which may be related to less adequate tissue blood perfusion at the relatively distally located stoma areas.

Individuals treated with an OFI-C experienced the least amount of infectious soft tissue adverse events, compared to OFI-Y and OTI, while individuals treated with a tibial OI encounter the most problems as is seen by comparing the infection/implant-year ratio. In our entire cohort we report an incidence of grade 1, 2, 3 and 4 of 13%, 11%, 0% and 1%, respectively. This differs when compared to infection rates in individuals treated with an OPL previously presented by Al Muderis et al. with an incidence of grade 1, 2, 3 and 4 infection being 45%, 9%, 0% and 0%, respectively. [10] This contrast might be explained by differences in in- and exclusion criteria as we report on a case-mix of individuals with a transfemoral and transtibial amputation and also included individuals with a dysvascular cause of amputation. Inter-rater variability with the use of a non-validated grading system might also influence the differences in outcome between studies. To this date, adverse events occurring in individuals treated with an OTI are typically under-reported as was stated in a review by Atallah et al. [15]. Serious adverse events that were reported were aseptic loosening: 29%, grade 4 implant infection: 29% and explantation: 43%. These disappointing results are in strong contrast with the results of tibial OI presented in this study in which one individual with dysvascular amputation developed grade 4 implant infection (5% of OTI). Better patient selection, improved surgical technique, implant design and better understanding of daily loading profile might play a key role in reducing adverse events associated with OTI treatment.[25]

Although the number of infectious soft tissue events in the group treated with a tibial OI was higher compared to the group treated with a femoral OI, this did not affect the quality of life and prosthetic use scores in the tibial OI group. We assume that the temporary and mild aspect of the infectious soft tissue events ultimately have no effect on the quality of life and prosthetic use scores. Although one individual developed septic implant loosening, we suspect that this is related to comorbidity and aetiology of amputation. The fact that no other septic loosening occurred and that other infectious soft tissue events did not lead to implant loosening within the first year after OI surgery is a promising result and long-term follow-up studies are required to evaluate implant survival in the longer term.

There are limitations associated with this study. First, the short follow-up period of one year precludes us from definitive conclusions with regard to implant survival on a long term. Second, adverse events were collected retrospectively based on patient reports, and no general practitioners were contacted; which may lead to an underestimation of the total number of adverse events. Third, the infectious complications were graded using an earlier developed system by Al Muderis et al. (S3 Appendix), which is not validated and thus may lead to inter-rater variability. Fourth, a subset of individuals was treated with single stage surgery while the rest was treated with two-stage surgery. This may have led to misinterpretation of the results, while there is still a lack of knowledge with regards to the safety of single stage surgery, especially in individuals with a transtibial amputation.[26] Fifth, there is little insight in confounders such as loading during daily living or alignment of components used, possibly associated with certain adverse events; such as the four DCA breakages that occurred.[25] Earlier research in individuals treated with screw-type implants revealed potential limitations of load monitoring, differences in loading compliance, and benefits of using certain instruments to monitor static load bearing.[27–29]

Future research should be performed to gain more insight in the effects of load bearing, during the rehabilitation time and in daily living with regard to adverse events such as component breakages and the effects of modifications of soft tissue surgical technique of the stoma with regard to soft tissue adverse events.

## Conclusion

This study shows that press-fit OI’s can be safely used in individuals with different levels of amputations, leading to an improvement in performance and acceptable complication rates at 1-year follow-up. These results may contribute to inform individuals with a lower extremity amputation and medical professionals of the risks and benefits of OI treatment so they can make an educated choice. Additional research with longer follow up period is required and currently on-going.

## Supporting information

S1 Appendix STROBE Checklist. (PDF)

S2 Appendix Clinical pathway. (PDF)

S3 Appendix Classification of infection. (PDF)

S1 File SPSS files of data. (SAV)
