# Supplementary material for: Safety, prosthesis wearing time and health-related quality of life of lower extremity bone-anchored prostheses using a press-fit titanium osseointegration implant: A prospective one-year follow-up cohort study
Source: PLoS One. 2020 Mar 9;15(3):e0230027. doi: 10.1371/journal.pone.0230027 (PMC7062258; doi:10.1371/journal.pone.0230027)
Supplement: S2 Appendix — (PDF) [file pone.0230027.s002.pdf]

S2 Appendix: Clinical pathway

Pre-surgical planning

CT scans and antero-posterior (AP) X-rays with calibration ball/ruler were used for pre-surgical planning and to define the right size of the intramedullary component of the osseointegration implant (OI). For an OFI-C the minimum length of the femoral remnant has to be 160mm, for an OFI-Y 40mm, from the mid lesser trochanteric line caudally. (Fig. 1, 2). For an OTI the minimal length of the tibial remnant has to be 60mm below the tibial plateau (Fig. 3)

For individuals with a longer femoral remnant, a calibrated AP full leg length conventional radiograph was used to calculate the desired femur length and optional shortening to equalize with the contralateral knee axis. For generally available prosthetic knee joints, the distance between the tip of the femur and the contra-lateral knee joint space should be at least 140mm (Fig. 4).

For individuals with a longer tibial remnant the distance between the tip of the tibia and the tibial plafond should be at least 170mm to fit generally available prosthetic feet.

The size of the DCA was estimated based on the thickness of the subcutaneous fat layer with the aim for at least 20mm of titanium niobium nitride (TiNbN) coating of the DCA to penetrate through the skin (Fig. 4).

Surgical procedure

OI surgery was performed under general or spinal anaesthesia including prophylactic intravenous antibiotics cephazolin (2g) at induction of anaesthesia. The patient was placed in supine position on a radiolucent operation table. Draping and prepping was performed in a fashion similar to that used for standard total hip or knee replacement. During the first surgery the medullary canal preparation involved reaming in a stepwise fashion with radiographic guidance to obtain cortical press-fit contact with the intramedullary component of the OFI or OTI. For OFI-C, diaphyseal reaming started with flexible reamers and was followed by OFI-C-specific curved reamers with a 2000mm radius with 1mm

increments depending on the bone quality. For OFI-Y and OTI the medullary diaphyseal canal preparation was achieved with rigid drills with either 1mm increments or not using power tools. Soft tissue surplus at the level of the distal stump was resected when indicated and the wound subsequently closed. Myodesis was performed only in OFI-C cases with burr holes at the distal end of the femoral remnant. During the second stage surgery a transcutaneous connection to the intramedullary component was created with a coring device (‘stoma’) and the DCA of the OFI/OTI was mounted to the intramedullary component.

Osseointegration Femur Implant: Curved type (OFI-C)

The OFI-C is a slightly curved (radius 2000mm) intramedullary component used for femoral remnants with a length of at least 160 mm measured starting from the mid-lesser trochanter line (Fig. 1). The non-tapered stem of the OFI-C is curved to match the anatomical antecurvation of the femur diaphysis. The OFI-C is manufactured by CNC milling from titanium (ISO 5832-11 Ti6Al7Nb). The extramedullary distal head is mirror polished and TiNbN coated creating a smooth surface allowing free movement of soft tissue at the stoma. The distal 70mm of the intramedullary stem is coated with plasma sprayed titanium (TPS) which promotes early osseointegration. The proximal part of the OFI-C stem is grit blasted also allowing osseointegration and has a portion with 10 flutes in longitudinal direction providing initial rotational stability which was adopted from the Wagner-shape femoral hip stems.(1) The OFI-C is available in two types depending on the length and diameter: the OFI-C OPL are manufactured by Permedica SPA (Merate, Italy) with a length of 160mm and diameters of 14 to 22mm with 1mm increments while the OFI-C OFP has a length of 140mm and diameter of 15 to 22mm with 1mm increments being manufactured by OTN Implants BV (Arnhem, the Netherlands). The OPL and OFP are identical regarding material, design, coating and surgical technique but the OFP is 20mm shorter than the OPL thus providing a larger application area because of the possible use in individuals with shorter femoral remnants.

Osseointegration Femur Implant: Gamma type (OFI-Y)

The OFI-Y is a straight intramedullary component indicated to be used in individuals with short femoral remnants, being less than 160mm from the mid-minor trochanteric line to the tip of the femur (Fig. 2). The OFI-Y is manufactured by direct metal laser sintering (DMLS) three-dimensional (3D) printing technology from titanium powder (ISO 5832-11 Ti6Al7Nb). The OFI-Y stem has a 1mm thick 3D lattice structure allowing for early bony ingrowth, while the extramedullary distal head is mirror polished and TiNbN coated. The proximal part of the stem has an 125 degrees oblique 10.5mm diameter hole (caput-collum-diaphyseal angle) which can be used to add a 10.45mm titanium cannulated lag screw through the implant into the femoral head. This lag screw fixation can be used as an option to provide maximal primary stability and may prevent stress fractures of the femoral neck in the long term. The length of the OFI-Y stem varies from 80 to 140mm with 10mm increments and the diameter varies from 16 to 23mm with 1mm increments. The OFI-Y used in this study is the OFP manufactured by OTN Implants BV (Arnhem, the Netherlands).

Osseointegration Tibia Implant (OTI)

The OTI is a straight intramedullary component for the tibial remnant (Fig. 3). The OTI is manufactured by direct metal laser sintering 3D printing technology from titanium powder (ISO 5832-11 Ti6Al7Nb). The OTI stem has a 1mm thick 3D lattice structure allowing for early osseointegration similar to the OFI-Y. The distal head is mirror polished and TiNbN coated. The proximal part of the stem has two 5.0mm diameter holes which can be used to insert 5.0mm transverse locking screws. The OTI stem fans out in a drop like shape distally to provide an adequate sealing of the intramedullary space of the tibia (Fig. 5). The drop-like shape portion of the OTI stem is grit blasted to allow osseointegration. The length of the OTI stem varies from 60 to 100mm with 10mm increments and the diameter from 17 to 30mm with 1mm increments. The OTI used in this study is the OTP manufactured by OTN Implants BV (Arnhem, the Netherlands).

Dual cone adapter and locking screw (DCA)

The DCA is a cylindrical transcutaneous component that is attached to the 16/18 taper of the intramedullary component of the OI with a M6 locking screw (Fig. 6). The DCA has two 16/18mm morse tapers. The proximal taper connection with the intramedullary component is provided with two weakpoints which are indicators for a proper connection. The weakpoints will break when the taper connection unexpectedly becomes loose during daily activities or as a result of high rotational impact forces as part of a safety mechanism protecting the bone and intramedullary implant. The distal taper of the DCA has a M14 thread which can accommodate a M14 abutment screw. The DCA and locking screw are manufactured by CNC milling and are made from titanium (ISO 5832-11 Ti6Al7Nb). The cylindrical part of the DCA is mirror polished and TiNbN coated. The DCA is available in 5 sizes, varying in length from 70 to 110mm with 10mm increments; and with a diameter of 18mm. The DCA and locking screws are manufactured by Permedica (Merate, Italy) and OTN Implants BV (Arnhem, the Netherlands).

Osseointegration implant connector

The osseointegration connector is a quick release-attach system for connecting the prosthetic parts to the DCA. It comprises a male and female part. The male part of the connector is attached to the distal DCA taper with a M14 abutment screw. The female part of the connector contains a clamp mechanism for quick attach and release fixation to the connector male part. The OI connector is provided with different and adjustable off-set sizes. The artificial limb is attached to the OI connector with a universal pyramid adapter. OI connectors used in this study are manufactured by OTN BV (Wychen, the Netherlands) and Hermle GmbH (Gosheim, Germany) (Fig. 7).

Prosthetic components and alignment

All individuals started their rehabilitation with the same prosthetic components as prior to the OI surgery, there were no specific component requirements for inclusion. The socket was removed and replaced by an OI connector. Prosthetic components used at baseline and at one-year follow-up can be found in Table 1. Based on our clinical experience we optimized the alignment using the LASAR

Posture system (Otto BocK GmbH, Germany). The alignment in frontal plane was adjusted to provide a narrow base of support with the aim to decrease the patients' effort to position the center of gravity above the center of pressure during single leg stance without an ipsilateral bending of the trunk. In transfemoral BAP, ideally a valgus angle was applied in the pyramid adapter of the OI connector to position the femoral remnant in adduction so that the abductor muscles are able to work more physiologically. In case of the presence of an abduction contracture we adjusted the alignment step-by step from a varus alignment to a valgus alignment. Depending on the degree of hip flexion contracture, an offset of 0 to 60mm in the sagittal plane was applied below the OI connector. Depending on the decrease in hip flexion contracture in the first year after OI surgery, the offset was gradually reduced. For transtibial BAP, the foot was positioned more medially or lateral using a sliding adapter based on clinical signs of excessive valgus or varus stress in the knee. In the sagittal plane an off-set of 0-20mm was usually applied in individuals with an OTI to facilitate a yielding in the early stance phase.

Table 1: Prosthetic component data

	**Baseline**		**One-year follow-up**	
	**Prosthetic knees (MPK vs non-MPK)***	**Prosthetic feet (ESAR vs non-ESAR)***	**Prosthetic knees (MPK vs non-MPK)***	**Prosthetic feet (ESAR vs non-ESAR***
**OFI-C (n=53)**	28 MPK , 18 non-MPK	40 ESAR, 6 non-ESAR	35 MPK, 18 non-MPK	48 ESAR, 5 non-ESAR
**OFI-Y (n=16)**	6 MPK, 2 non-MPK	7 ESAR, 1 non-ESAR	9 MPK, 7 non-MPK	15 ESAR, 1 non-ESAR
**OTI (n=21)**	NA	19 ESAR	NA	17 ESAR, 2 non-ESAR

MPK: Microprocessor knee, ESAR: Energy storing and return, OFI-C: Osseointegration Femur Implant curved type, OFI-Y Osseointegration Femur Implant Gamma type, OTI: Osseointegration Tibia implant. N: total number of individuals in cohort subgroup, * Number of individuals using a prosthesis at certain time point.

References

1. Wagner SL Revision®Hip. http://wwwzimmernl/medical-professionals/products/hip/wagner-sl-revision-hiphtml.
