# Supplementary material for: Safety, prosthesis wearing time and health-related quality of life of lower extremity bone-anchored prostheses using a press-fit titanium osseointegration implant: A prospective one-year follow-up cohort study
Source: PLoS One. 2020 Mar 9;15(3):e0230027. doi: 10.1371/journal.pone.0230027 (PMC7062258; doi:10.1371/journal.pone.0230027)
Supplement: S3 Appendix — (PDF) [file pone.0230027.s003.pdf]

S3 Appendix: Classification of infection

Table 1. Classification of infection			
Level of Severity	Symptoms and Signs	Treatment	Grade
Low-grade soft tissue infection	Cellulitis with signs of inflammation (redness, swelling, warmth, stinging pain, pain that increases on loading, tense)	- Oral Antibiotics<br>- Parenteral Antibiotics<br>- Surgical Intervention	1<br>1A<br>1B<br>1C
High-grade soft tissue infection	Pus collection, purulent discharge, raised level of C-reactive protein	- Oral Antibiotics<br>- Parenteral Antibiotics<br>- Surgical Intervention	2<br>2A<br>2B<br>2C
Bone infection	Radiographic evidence of osteitis (periosteal bone reaction), radiographic evidence of osteomyelitis (sequestrum and involucrum)	- Oral Antibiotics<br>- Parenteral Antibiotics<br>- Surgical Intervention	3<br>3A<br>3B<br>3C
Implant failure	Radiographic evidence of loosening	- Parenteral antibiotics, explantation	4
